# Notes on Treatment

**Published:** 1908-05-02

**Authors:** 


### NOTES ON TREATMENT.

### The Dangers of Formalin.

Formalin is of value as an antiseptic, as an external application in ringworm and to allay itching, but it also has its inconveniences. Some people are curiously susceptible to its influence. In these even the vapour may produce attacks of eczema. Happily the susceptibility is one that is generally in a great degree acquired, that is to say those who suffer do not suffer when first brought into contact with formalin. When, however, one attack of eczema has occurred, the presence of the vapour, as already mentioned may be sufficient to excite a return of the inflammation.

### Incontinence of Urine in Children.

Sulphonal is a drug which may be used with caution and not uncommonly is successful in curing this troublesome complaint when other means have failed. Five, ten, or perhaps fifteen grains, according to the age of the patient, may be given at bedtime for a week, and then if there has been no nocturnal incontinence the same dose may be given every other night for the following week. During the third week the drug may be given with two nights intervening, and so on until it is dropped altogether. Trional, which is said to be a safer drug than sulphonal, apparently has little effect upon nocturnal enuresis.

